# Development and Disease: How Susceptibility to an Emerging Pathogen Changes through Anuran Development

**DOI:** 10.1371/journal.pone.0022307

**Published:** 2011-07-22

**Authors:** Nathan A. Haislip, Matthew J. Gray, Jason T. Hoverman, Debra L. Miller

**Affiliations:** 1 Department of Forestry, Wildlife, and Fisheries, Center for Wildlife Health, University of Tennessee, Knoxville, Tennessee, United States of America; 2 Veterinary Diagnostic and Investigational Laboratory, College of Veterinary Medicine, University of Georgia, Tifton, Georgia, United States of America; University of Bern, Switzerland

## Abstract

Ranaviruses have caused die-offs of amphibians across the globe. In North America, these pathogens cause more amphibian mortality events than any other pathogen. Field observations suggest that ranavirus epizootics in amphibian communities are common during metamorphosis, presumably due to changes in immune function. However, few controlled studies have compared the relative susceptibility of amphibians to ranaviruses across life stages. Our objectives were to measure differences in mortality and infection prevalence following exposure to ranavirus at four developmental stages and determine whether the differences were consistent among seven anuran species. Based on previous studies, we hypothesized that susceptibility to ranavirus would be greatest at metamorphosis. Our results did not support this hypothesis, as four of the species were most susceptible to ranavirus during the larval or hatchling stages. The embryo stage had the lowest susceptibility among species probably due to the protective membranous layers of the egg. Our results indicate that generalizations should be made cautiously about patterns of susceptibility to ranaviruses among amphibian developmental stages and species. Further, if early developmental stages of amphibians are susceptible to ranaviruses, the impact of ranavirus epizootic events may be greater than realized due to the greater difficulty of detecting morbid hatchlings and larvae compared to metamorphs.

## Introduction

Disease epidemics are driven by the complex interactions among the pathogen, host susceptibility, and the environment. Recent work in disease ecology seeks to understand mechanisms of pathogen infection during development that lead to developmental abnormalities and mortality events [1]. There is increasing awareness that there are critical windows during development in which hosts are particularly sensitive to disease-causing agents leading to mortality, impairment, or malformation of the individual [1], [2]. In humans, for example, differences in susceptibility to infection during development are demonstrated by the early childhood malformations and mortality associated with German measles (Rubella Virus; [3]). Such developmental perturbations can occur from exposure to toxins, parasites, and nutrient deficiencies [1], [2], [4]. Thus, the connection between windows of developmental sensitivity and susceptibility to pathogens is an important mechanism in the emergence of wildlife diseases.

The role of pathogens in the recent declines of amphibians across the globe has received considerable attention [5]. While amphibians are hosts for a diversity of pathogens [6], many die-off events have been associated with infection by ranaviruses [7], [8]. Ranaviruses have been reported on five continents and are associated with nearly 50% of the reported amphibian mortality events in the United States [7], [9]. Although ranaviruses have been well studied and characterized at the molecular level [10], [11], research has only recently begun to examine the mechanisms associated with ranavirus emergence in wild populations [12].

In 96% of reported ranavirus die-off events, recently metamorphosed individuals experienced the greatest mortality [7], [9]. These field observations have led to the hypothesis that ranavirus epizootics in the wild occur most often as amphibians undergo metamorphosis, which is known to be a period of natural immune suppression [12]. Previous studies suggest that there are varying degrees of immune system development across different amphibian life stages. Du Pasquier et al. [13] found that the production of thymic lymphocytes increases during larval development, drops substantially at metamorphosis, and peaks in adult *Xenopus laevis*. Decreases in immune function during metamorphosis are probably related to endogenous production of glucocorticoids associated with restructuring organ systems for postmetamorphic life [14]. Thus, the immunological changes that occur during anuran development should affect host-pathogen interactions [14], [15]. Unfortunately, experimental studies comparing the susceptibility of amphibians to pathogens at different developmental stages are rare [15], [16]. Thus, the first objective of our research was to test for differences in susceptibility (as indexed by mortality and infection prevalence) to ranavirus among pre-terrestrial developmental stages in amphibians.

Traditionally, disease ecology has focused on pathogens that attack a single host, which has limited our ecological understanding of disease dynamics driven by pathogens that infect multiple host species [17]–[19]. There is growing evidence that amphibian species differ in their susceptibility to pathogens [20]–[23]. While not surprising, such variation in species susceptibility underscores the need for comprehensive studies that examine multiple host species to identify generalities that cannot be obtained from single-species studies. To date, very few studies have examined the relative susceptibility of amphibian larvae to ranaviruses [22], [24], [25], [26]. Moreover, these studies tested only one developmental stage, thus their results may be limited. The second objective of our study was to identify trends in the relative susceptibility to ranavirus for seven North American anuran species.

## Methods

### Ethics statement

All animal husbandry and euthanasia procedures followed an approved University of Tennessee IACUC protocol (#1755).

### Animal collection and maintenance

We used seven anuran species for our study: *Lithobates clamitans*, *L. pipiens*, *L. sylvaticus*, *Pseudacris feriarum*, *Hyla chrysoscelis*, *Scaphiopus holbrookii*, and *Anaxyrus americanus*, which are widely distributed in eastern North America [27]. Between February–July 2009, we collected 7–20 egg masses for each species from single populations (Table 1). Egg masses were collected within 48 hours of deposition, rinsed with sterile water, and transported in 19-L buckets filled with aged tap water to the University of Tennessee Joe Johnson Animal Research and Teaching Unit (JARTU). Egg masses were placed in covered (60% shade cloth) 300-L wading outdoor pools the day after collection to develop. After hatching, tadpoles were maintained in these pools and fed rabbit chow (Purina, St. Louis, Missouri) and ground TetraMin® (Tetra, Blacksburg, Virginia) *ad libitum* until used in the experiments. The experiments began as individuals reached the appropriate developmental stages (see below). Prior to each experimental trial, a random sample of 10 tadpoles was euthanized and frozen at −80°C for confirmation that they were negative for ranavirus using real-time quantitative polymerase chain reaction (qPCR, see Molecular Analyses section); all pre-experiment individuals tested negative.

**Table 1:** Quantity of egg masses and collection sites in Tennessee and Pennsylvania, USA.

Scientific Name	State	County	Location	Lat – Long	UTM	Quantity
*Anaxyrus americanus*	PA	Crawford	Pymatuning State Park	41°34′10″N, 80°27′20″W	17 545392E 4602117N	10
*Hyla chrysoscelis*	TN	Knox	Private landowner	36°01′30″N, 83°47′30″W	17 248426E 3990338N	9
*Lithobates clamitans*	TN	Union	Chuck Swan WMA	36°21′29″N, 83°54′49″W	17 238539E 4027616N	7
*Lithobates pipiens*	PA	Crawford	Pymatuning State Park	41°41′30″N, 80°30′20″W	17 541146E 4615661N	10
*Lithobates sylvaticus*	TN	Knox	Royal Blue WMA	36°02′10″N, 83°51′19″W	17 242745E 3991727N	9
*Pseudacris feriarum*	TN	Knox	Seven Islands Wildlife Refuge	35°56′59″N, 83°41′41″W	17 256940E 3981756N	20
*Scaphiopus holbrookii*	TN	Union	Chuck Swan WMA	36°21′29″N, 83°54′49″W	17 238539E 4027616N	20

### Virus isolate

A single isolate of *Ranavirus* was used for all experiments. The University of Georgia Veterinary Diagnostic and Investigational Laboratory (VDIL) extracted this isolate from morbid *L. catesbeianus* juveniles. Preliminary molecular analyses suggest that the isolate is similar to the ranavirus *frog virus 3* (GenBank accession no. EF101698, [28]), and it has been shown to be virulent in anuran larvae [22]. Titrated stock solutions of the isolate were sent overnight by the VDIL to the University of Tennessee for the experiments.

### Experimental protocol

For each species, we conducted a 14-d experimental trial for each of four developmental stages: 1) embryo (stage 11), 2) hatchling (stage 21), 3) larval (stage 30), and 4) pro-metamorphosis (stage 41, [29]). For our experiments, embryos were contained in eggs. Experimental units for all trials were 1-L tubs filled with 0.5 L of aged tap water. The tubs were placed at a common shelf height in a completely randomized design at the JARTU laboratory facility. We randomly assigned a single individual to each tub. Treatments included a no-virus control and a virus exposure of 10^3^ plaque-forming units (PFUs) mL^−1^ [22]. Both treatments were replicated 20 times for a total of 40 experimental units per trial.

We inoculated the water (i.e., bath exposure) with 29.5 µL of Eagle's Minimal Essential Media (MEM) for the no-virus control tubs and 29.5 µL of MEM containing the virus for the virus tubs. The resulting virus concentration was 10^3^ PFUs mL^−1^, which is within the range of doses used in other studies (10^2^–10^6^ PFUs mL^−1^; [30]–[32]) and ecologically relevant [24], [33]. Given that some species in our study developed rapidly (e.g., *S. holbrookii*), we used a 3-day exposure in an attempt to target the intended developmental stage rather than a subsequent stage. After three days, individuals were removed from the containers, rinsed with sterile water, and placed into a new container with 500-mL of fresh aged tap water. For the remainder of the experiment, water was changed every three days to maintain water quality.

After each water change, individuals in the larval and metamorph experiments were fed ground TetraMin® at a daily rate of 8% body mass [34]. Prior to the water change, we weighed a group of 10 non-experimental individuals housed under identical conditions to calculate food rations based on the average mass. Individuals in the embryo and hatchling experiments were fed if they reached stage 25 prior to the end of the experiment, which is when yolk reserves are exhausted and jaw development is complete in most species [35]. After the initial exposure and water change, platforms were placed in the metamorph experimental units to allow individuals to crawl out of the water to complete metamorphosis. Once individuals in the metamorph stage experiments began tail resorption, feedings were terminated and water depth was slowly reduced until a minimal amount of water remained to provide moisture for the individual and TetraMin® was no longer added. Following tail resorption, individuals were fed 10 seed weevils (*Callosobruchus sp.*) every three days.

The experimental units were monitored three times daily for mortality. Dead larvae and metamorphs were necropsied using sterilized forceps and scissors. Because the kidneys and liver are known sites of ranavirus infection [12], we removed sections of these organs from each individual, placed the pooled sample in a 1.5-mL microcentrifuge tube, and froze at −80°C for molecular testing. Dead embryos and hatchlings were rinsed with sterile water and frozen at −80°C, because their small size prevented consistent necropsies. After 14 days, all live individuals were euthanized in benzocaine hydrochloride (1 g L^−1^) and the identical necropsy procedures followed. We set 14 days as the experiment duration because previous research has shown this is sufficient duration to observe disease from ranavirus infection with a 3-day water bath exposure [22].

### Diagnostic testing

For ranavirus testing, genomic DNA (gDNA) was extracted from a homogenate of the kidney and liver for tadpoles and metamorphs and from entire embryos (including vitelline membrane and mucoidal capsules) and hatchlings using a DNeasy Blood and Tissue Kit (Qiagen Inc., Valencia, CA). We used the Qubit™ fluorometer and the Quant-iT™ dsDNA BR Assay Kit to quantify the concentration of genomic DNA in each sample (Invitrogen Corp., Carlsbad, CA, USA) [36]. The qPCR amplified a 70-bp region of the ranavirus major capsid protein. For each sample, we combined 12.5 µL of TaqMan Universal PCR Master Mix (Applied Biosystems, Foster City, California, USA), 1.5 µL of each primer (rtMCP-F [5′ – ACA CCA CCG CCC AAA AGT AC – 3′] and rtMCP-R [5′ – CCG TTC ATG ATG CGG ATA ATG – 3′]), and 1.5 µL of rtMCP-probe (5′-CCT CAT CGT TCT GGC CAT CAA CCA-3′). We added 0.25 µg of gDNA from each sample to standardize the total amount of gDNA added to the tubes. Because the volume containing this amount of gDNA varied depending on the gDNA concentration of the sample, we used the values from the fluorometer to calculate how much of the sample to add. We then added DNA grade water to the sample to bring the total volume to 30 µL. A SmartCycler® (Cepheid, Sunnyvale, California) thermal cycler was used for the qPCR. In each run of the qPCR, we included 4 controls, which were a ranavirus-negative tadpole sample, a negative DNA grade water sample, a ranavirus-positive tadpole sample, and a cultured virus sample. For each sample, we recorded the cycle number at which the sample crossed the fluorescent threshold level, which was set at 30 (i.e., CT value). Those samples that crossed the threshold level before CT = 30 were declared infected.

### Statistical analysis

The response variables for each experiment included final mortality and infection prevalence calculated from binary data. Differences in final mortality and infection prevalence were tested among species and developmental stages using logistic regression analysis [37], [38]. We did not include the control treatment in the analysis because control mortality was low resulting in low or zero counts for prevalence estimates of several developmental stages, which could have biased the logistic regression results [37], [38]. Instead, median control mortality among developmental stages was provided for each species. If the Wald's chi-square test associated with the logistic regression analysis was significant, we used binomial tests that were Bonferroni corrected (α÷number of post-hoc comparisons) to test for pairwise differences between proportions [38]. We estimated the likelihood of infection and mortality for each treatment in comparison with the treatment having the lowest rate by calculating odds-ratio statistics [37]. If species and developmental stage effects interacted, we separated the analysis by species and performed a chi-square test for differences in mortality and infection prevalence among stages. All tests were performed at α = 0.05 using PROC LOGISTIC in the SAS® system [37]. Test statistics and *P*-values were provided for evidence of differences in infection prevalence and mortality among effect levels. Test statistics with inequalities included results from more than one effect. Lastly, we regressed infection prevalence against mortality using linear regression in PROC GLM. Paired estimates for infection and mortality were the response variables and included in the analysis only if both proportions were not zero.

## Results

**Figure 1:**
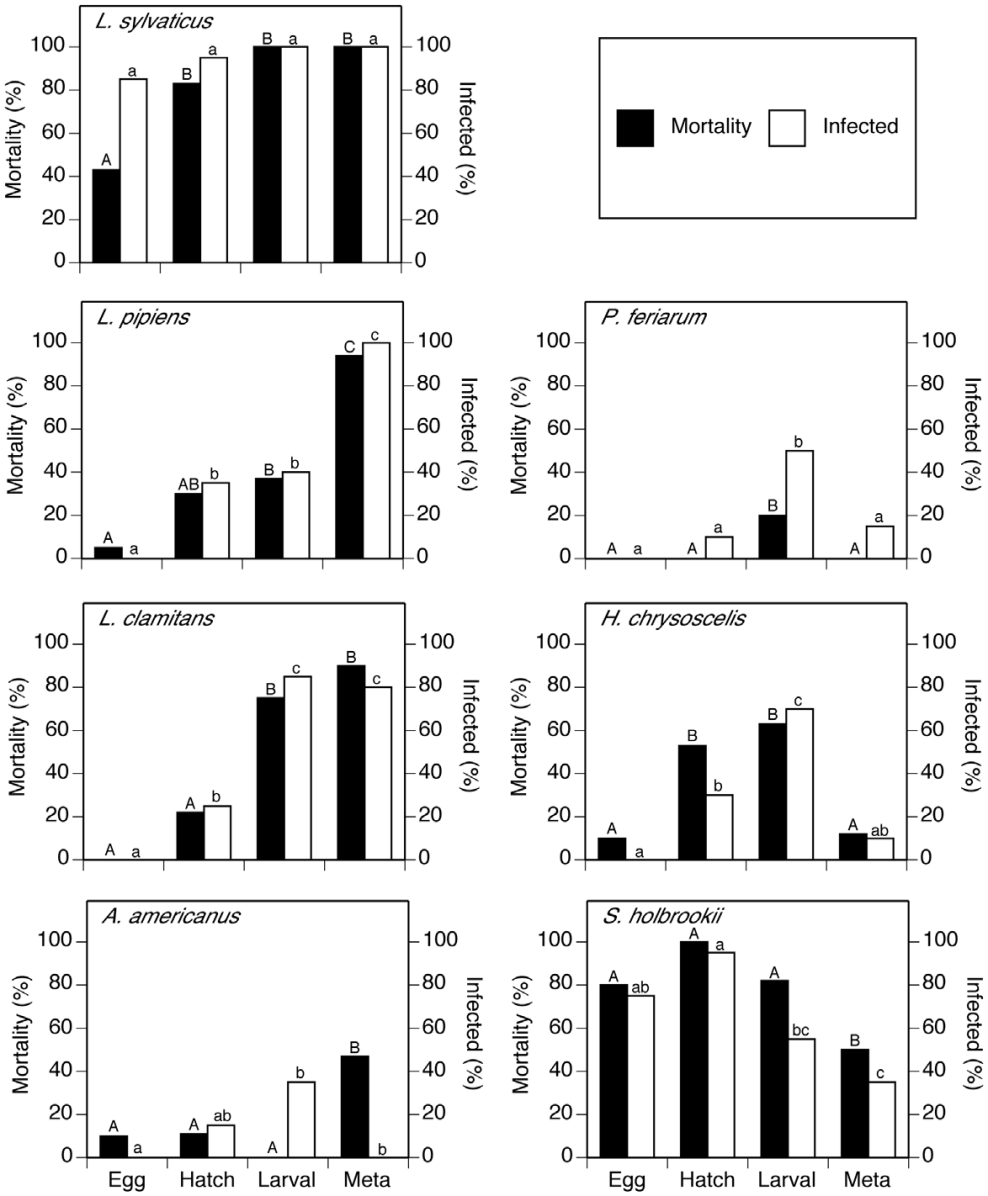
Percent mortality and infection among embryo, hatchling, larval, and metamorphosis developmental stages for *Lithobates sylvaticus*, *L. pipiens*, *L. clamitans*, *Anaxyrus americanus*, *Pseudacris feriarum*, *Hyla chrysoscelis*, and *Scaphiopus holbrookii*. Similar shaded bars with unlike letters are different (*P*<0.006) by logistic regression analysis; *n* = 20 per developmental stage for each species.

Across all species, final mortality and infection prevalence for the hatchling, larval and metamorph stages were significantly greater than the embryo stage (χ^2^_3_>43.3, *P*<0.001). In the hatchling, larval, and metamorph stages, the odds of mortality were 3X, 4X, and 5X greater, respectively, when exposed to ranavirus compared to the embryo stage. Across all developmental stages, mortality and infection were greatest for *L. sylvaticus* and *S. holbrookii*, and were lowest for *P. feriarum* and *A. americanus* (χ^2^_6_>40.67, *P*<0.001; Figure 1). Intermediate mortality and infection occurred for *L. clamitans*, *L. pipiens*, and *H. chrysoscelis* (Figure 1). Ranavirus exposed *L. sylvaticus* and *S. holbrookii* had 150X and 119X greater odds of mortality, respectively, than *P. feriarum*. Among species and stages, there was a strong positive relationship (*R* ^2^ = 0.79) between mortality and infection prevalence (*F*_1,20_ = 74.52, *P*<0.001).

Species and developmental stage effects interacted for final mortality and infection prevalence (χ^2^_18_ = 128.9, *P*<0.001); thus, logistic regression analyses were performed separately for each species. For all species except *L. sylvaticus*, mortality and infection prevalence differed among developmental stages (χ^2^_3_>12.6, *P*<0.006; Figure 1). For *L. sylvaticus*, infection prevalence was high (>82%) and did not differ among stages (χ^2^_3_ = 6.3, *P* = 0.09). Mortality and infection prevalence were greatest during the metamorph stage for all *Lithobates* species. Mortality also was greatest during the metamorph stage for *A. americanus*, but these individuals were not infected with ranavirus. Mortality and infection prevalence tended to be greatest during the larval stage for the two hylid species: *P. feriarum* and *H. chrysoscelis*. The greatest infection and mortality for *S. holbrookii* occurred during the embryo, hatchling and larval stages, and were lowest during metamorphosis. Median control mortality was low for all species (≤10%), except for *P. feriarum* (22.5%), thus the results for this species should be interpreted cautiously. No control tadpoles tested positive for ranavirus infection.

## Discussion

Embryos that were contained within eggs were the least susceptible stage across species when exposed to ranavirus in a water bath. Previous research has shown that direct injection of ranavirus into embryos causes 97–100% mortality in *L. pipiens* [39]. Thus, the vitelline membrane encasing the developing embryo or the mucopolysaccharide/mucoprotein capsules coating the surface of the egg likely affords protection against ranavirus infection. The mechanisms that contribute to this protection are unknown but may include structural barriers [40], [41] or anti-viral properties of the egg capsules or membrane [42]. Infection occurred in the embryo experiments for *S. holbrookii* and *L. sylvaticus*; however, embryos of these species hatched prior to the end of the 3-day virus challenge, hence exposing the hatchling to virions. No infection occurred during the embryo experiments in species that hatched after the virus challenge and first water change. Thus, it appears that eggs protect their developing embryos from ranavirus infection for the species we tested.

We documented high mortality during metamorphosis for all species of *Lithobates* tested, which is frequently the stage documented during anuran die-offs in the wild [43], [44]. Cullen et al. [25] and Cullen and Owens [26] reported high susceptibility of several species of recently metamorphosed anurans compared to larvae or adults when exposed to ranavirus. Warne et al. [45] also reported higher mortality of ranavirus-exposed *L. sylvaticus* tadpoles during metamorphosis. High infection and mortality during metamorphosis may be associated with decreased immune function from endogenous production of corticosteroids and lymphocyte apoptosis [14], [45], [46], [47], which has been demonstrated in *X. laevis* [48], [49].

All other species that we tested had low mortality and infection prevalence during metamorphosis. The classic model of amphibian immune function during development, based on *X. laevis*, suggests that immune function increases through development then drops during metamorphosis [47]. Down regulation of the immune system during metamorphosis may prevent destruction of new cell types that form for terrestrial life or may be a consequence of reduced physiological resources [14], [45]. According to the *X. laevis* model of immune function, mortality associated with ranavirus infection should have been lowest during the larval (i.e., tadpole) stages. Lowest mortality during the larval stage did not occur for any of the anuran species that we tested, which may indicate that immune responses of North American anurans differ from those of *X. laevis*. The fully aquatic life cycle of *X. laevis* may result in unique immunological adaptations that are not shared with amphibian species that live terrestrially after metamorphosis. Pallister et al. [50] suggested that differences in larval development might contribute to differences in immune function. Indeed, comparative immunological studies between *X. laevis* and other anuran species are needed.

The greatest mortality and infection prevalence occurred during the hatchling stage for *S. holbrookii*, which was a different trend among the species that we tested. Infection and mortality decreased during the larval and metamorph stages, suggesting that immune function increased through development for this species. Compromised immunity during early development may be a consequence of physiological trade-offs associated with rapid development in this species. Spadefoots are among the fastest developing anuran species due to their association with ephemeral breeding sites [51], [52]. Zettergren [53] reported cells synthesizing immunoglobulins (Ig) during embryogenesis and B lymphocytes circulating in pre-metamorphic *L. pipiens* at the onset of feeding. Leukocyte mobilization and anti-FV3 IgY antibody production have been reported as immune responses to ranavirus infection in *X. laevis* [54], [55]. We hypothesize that development of these components of the amphibian immune system is delayed in *S. holbrookii* due to rapid growth during the embryo and hatchling stages.

Among species, *L. sylvaticus* was the most susceptible, with infection and mortality exceeding 80% in the hatchling, larval, and metamorph stages. These results support field observations for this species across its geographic range [21], [44], [56], [57]. To date, no studies have explored the immunological mechanisms underlying the high susceptibility of *L. sylvaticus* to ranavirus compared to other species, although see Warne et al. [45]. Cotter et al. [58] reported that poor lymphocyte production in the spleen was a mechanism driving high susceptibility of larval *Ambystoma mexicanum* to ranavirus. Significant increases in total leukocytes and natural killer cells are detected after 1 and 3 days post-infection with ranavirus, respectively, in *X. laevis* [55]. Pre-metamorphic *L. catesbeianus* and *X. laevis* produce antibodies [59], [60], and therefore may resist ranavirus infection [61]. Thus, minimal innate and adaptive immune response to ranavirus infection may be mechanisms contributing to high infection and mortality rates in ranavirus-exposed *L. sylvaticus*.

Our study is the first to report mortality of anuran hatchlings by ranavirus. The possibility for hatchling mortality from ranaviruses raises a significant conservation concern considering that detecting die-offs of hatchlings is extremely difficult in the wild. Differential susceptibility among developmental stages also indicates that studies that focus on one stage [22], [24] may provide narrow insight into species susceptibility. If testing only one stage is feasible, we recommend using the larval stage because mortality and infection prevalence were either greater or similar to hatchling and metamorph stages for most species.

More research is needed investigating the role of immune function in regulating differences in susceptibility to ranavirus among anuran species. To date, few studies have quantified immune responses to ranavirus in pre-metamorphic amphibians [15], [58]. Identifying commonalities among immunogenetic, evolutionary and life history traits of susceptible species will improve our understanding of host-pathogen interactions [62], and help facilitate identification of amphibian communities at greatest risk of ranavirus epizootics. To this end, we recommend that additional amphibian species and ranavirus strains be tested for relative susceptibility. Various multivariate techniques exist (e.g., canonical correspondence analysis, [63]) that can elucidate patterns between host characteristics and indices of susceptibility. We also encourage studies that challenge amphibian species with ranavirus at each stage of development and follow individual survival through metamorphosis. This knowledge is fundamental to developing stage-structured disease models that predict epizootic outcomes [64].
